# Implementation of a Cellulitis Management Plan in Three Australian Regional Health Services to Address an Evidence–Practice Gap in Antibiotic Prescribing

**DOI:** 10.3390/antibiotics10111288

**Published:** 2021-10-22

**Authors:** Jaclyn Bishop, Mark Jones, James Farquharson, Kathrine Summerhayes, Roxanne Tucker, Mary Smith, Raquel Cowan, N. Deborah Friedman, Thomas Schulz, David Kong, Kirsty Buising

**Affiliations:** 1 National Centre for Antimicrobial Stewardship (NCAS), Peter Doherty Research Institute for Infection and Immunity, The University of Melbourne, Level 5, 792 Elizabeth Street, Melbourne, VIC 3000, Australia; thomas.schulz@mh.org.au (T.S.); david.kong@bhs.org.au (D.K.); Kirsty.Buising@mh.org.au (K.B.); 2 Faculty of Medicine, Dentistry and Health Sciences, Department of Medicine- RMH, The University of Melbourne, Royal Parade, Melbourne, VIC 3000, Australia; 3 Pharmacy Department, Ballarat Health Services, Drummond Street North, Ballarat, VIC 3350, Australia; Mark.Jones@bhs.org.au; 4 Pharmacy Department, Colac Area Health, Connor Street, Colac, VIC 3250, Australia; JFarquharson@cah.vic.gov.au; 5 Clinical Improvement, Risk and Innovation, Wimmera Health Care Group, Baillie Street, Horsham, VIC 3400, Australia; Kathrine.Summerhayes@whcg.org.au (K.S.); roxanne.tucker@whcg.org.au (R.T.); 6 Department of Health and Human Services (Victoria), McLachlan Street, Horsham, VIC 3000, Australia; Mary.Smith@dhhs.vic.gov.au; 7 Department of Internal Medicine, Ballarat Health Services, Drummond Street North, Ballarat, VIC 3350, Australia; Raquel.Cowan@bhs.org.au; 8 Department of Infectious Diseases, Barwon Health, Ryrie Street, Geelong, VIC 3220, Australia; deborahf@barwonhealth.org.au; 9 School of Medicine, Faculty of Health, Deakin University, Pigdons Road, Waurn Ponds, VIC 3216, Australia; 10 Victorian Infectious Diseases Service, The Royal Melbourne Hospital, 300 Grattan Street, Melbourne, VIC 3050, Australia; 11 Department of Infectious Diseases, Peter Doherty Institute for Infection and Immunity, The University of Melbourne, 792 Elizabeth Street, Melbourne, VIC 3000, Australia; 12 Centre for Medicine Use and Safety, Monash Institute of Pharmaceutical Sciences, Monash University, 381 Royal Parade, Parkville, VIC 3052, Australia

**Keywords:** cellulitis, antibiotic, stewardship, appropriateness, rural

## Abstract

Despite the availability of evidence-based guidelines, antibiotics for cellulitis remain inappropriately prescribed. This evidence–practice gap is more evident in low-resource settings, such as rural hospitals. This implementation study developed and introduced a cellulitis management plan to improve antibiotic prescribing for cellulitis in three health services in regional Australia. Appropriateness of antibiotic prescribing for cellulitis at Day 1 was the primary outcome measure. Adults with ICD-10-AM codes for lower-limb cellulitis admitted as inpatients of the three health services between May and November 2019 (baseline, *n* = 165) and March and October 2020 (post-implementation, *n* = 127) were included in the assessment. The uptake of the cellulitis management plan was 29.1% (37/127). The appropriateness of antibiotic prescribing for cellulitis at Day 1 was similar at baseline (78.7%, 144/183) and in the intention-to-treat post-implementation group (81.8%, 126/154) [95% CI −5.6% to 11.3%, *p* = 0.50]. Commencement of the cellulitis management plan resulted in a non-statistically significant increase in antibiotic appropriateness at Day 1 compared to when a cellulitis management plan was not commenced (88.1% vs. 79.5%; 95% CI −5.6% to 19.8%; *p* = 0.20) Evaluation of more real-world strategies to address evidence–practice gaps, such as the appropriateness of antibiotic prescribing for cellulitis, is required.

## 1. Introduction

Cellulitis is a common condition that consumes health service resources. A retrospective cohort analysis of US hospital admissions in 2014 reported that 9.8% of over 447,000 cellulitis admissions were associated with non-elective readmission within 30 days at an estimated cost of USD 500 million [1]. In Australia, in 2017–2018, there were 68,664 hospital separations for cellulitis, equating to 258 hospitalisations per 100,000 people [2]. Of even greater concern, cellulitis contributed 9% of all potentially preventable hospitalisations in Australia, and this rate varied depending on geographical location [2].

Inappropriate antibiotic choices for cellulitis are a commonly reported outcome internationally. A recent study of antibiotic prescribing for patients admitted with cellulitis to an Irish district general hospital concluded that there was a significant discrepancy between current clinical practice and international guidelines for the management of cellulitis [3]. A study in 34 Veterans Affairs Medical Centers in the USA that evaluated antibiotic prescribing for skin and soft tissue infections (cellulitis and abscess) reported that only 14% of patients received guideline-concordant empiric therapy and an appropriate duration of therapy [4]. In Australia, the National Antimicrobial Prescribing Survey (NAPS, a point prevalence survey of Australian hospitals) indicated that cellulitis/erysipelas was the fifth most common indication for prescribing an antibiotic in public and private hospitals, with an appropriateness of antibiotic choice at 75.3% [5]. Additionally, an analysis of NAPS data between 2014 and 2016 indicated that antibiotics for cellulitis were more often prescribed inappropriately in Australian regional and remote hospitals compared to Australian major-city hospitals (25.7% vs. 19.0%, *p* ≤ 0.001) [6]. This is despite the availability of Australian evidence-based guidelines on antibiotic prescribing for cellulitis [7].

These data highlight the need to improve the implementation of evidence-based recommendations for cellulitis care and to ensure that the strategies adopted are suitable for the regional healthcare setting. This study describes the co-production, implementation, and evaluation of a novel cellulitis bundle and management plan intended to influence antibiotic prescribing practices in three regional health services in Victoria, Australia. 

## 2. Results

**Figure 1:**
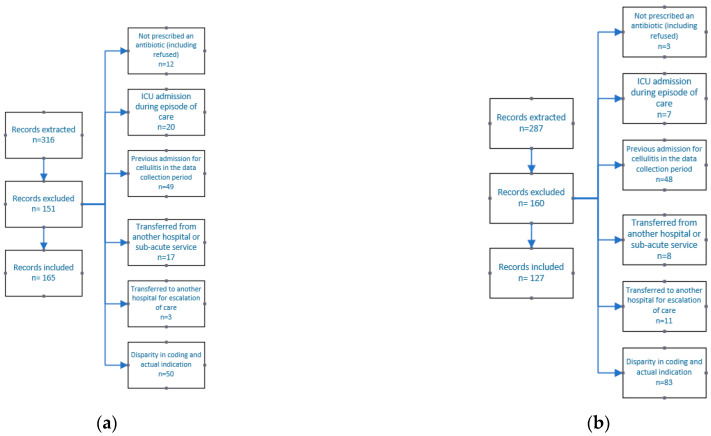
Reasons for exclusion from the sample: (**a**) baseline (*n* = 165) and (**b**) post-implementation sample (*n* = 127).

There were 165 patients included in the baseline and 127 in the post-implementation audit of antibiotic appropriateness. Reasons for exclusion are shown in Figure 1.

The two groups were similar in their demographics and clinical parameters at presentation (Table 1). While temperature, heart rate, and respiratory rate were statistically higher in the post-implementation group, these were not considered clinically significant differences. 

The number of eligible patients who had a cellulitis management plan initiated, as evidenced by a scanned copy of the cellulitis management plan in the patients’ medical records, was 29.1% (37/127). Antibiotic appropriateness at day 1, day 3, and discharge was similar in the baseline and post-implementation groups (Table 2). Commencement of the cellulitis management plan resulted in a non-statistically significant increase in antibiotic appropriateness at day 1, day 3, and discharge compared to when a cellulitis management plan was not commenced. The reasons for why the prescriptions were deemed inappropriate are outlined in Table S1.

Flucloxacillin and cefazolin were the two most prescribed antibiotics on Day 1 at baseline and post-implementation. There were fewer orders for piperacillin/tazobactam in the post-implementation group and more orders of benzylpenicillin (Table 3).

There were no statistically significant differences between baseline and post-implementation for the secondary outcome measures (presentation to the Emergency Department, readmission within 30 days of discharge with cellulitis, acute length of stay, duration of total, inpatient, or IV antibiotic therapy), nor between those that had the cellulitis management plan commenced and those that did not (Table 4). The median time to first antibiotic dose significantly decreased in the post-implementation period, but a statistically significant difference was not observed between those that had the cellulitis management plan commenced and those that did not (Table 4).

The limb elevation plan was the most completed section of the cellulitis management plan (24, 64.9%), followed by the antibiotic choice (18, 48.6%) and location of care (14, 37.8%). Obesity (10) and the presence of a wound (9) were the most common predisposing factors identified in the cellulitis management plan. There were 15 patients who had no predisposing factors marked.

Interviews were completed with two Emergency Department physicians, a pharmacist, a wound-care consultant, and a nurse providing parenteral therapies in the home. Pertinent quotes are listed Table 5. There were positive views on the usefulness of the cellulitis management plan. Respondents described it as a working tool that provided clear prescribing advice, addressed risks, and communicated expectations. One respondent suggested that it did not add value to their practice because they were experienced in managing cellulitis. There were encouraging comments about the impact of the cellulitis management plan on practice. Respondents indicated that they learnt about changes in prescribing guidance (such as the use of benzylpenicillin first line for cellulitis likely caused by Streptococcus) [7] by using the cellulitis management plan. Even in the absence of a cellulitis management plan being commenced for a patient, an influence on practice was described.

The challenges in creating a resource that was comprehensive and multidisciplinary were evident in the comments around the bulkiness of the cellulitis management plan. Doctors identified more readily with the earlier sections of the cellulitis management plan that supported diagnosis and antibiotic choice. There were mixed views on who should be responsible for completing the cellulitis management plan. Responsibility was described as often defaulting to nurses in similar initiatives. However, the diagnostic and antibiotic choice components of the cellulitis management plan were thought to align with the scope of practice for a doctor. Pharmacists were identified as another profession with potential scope, but not capacity, to initiate the cellulitis management plan. Other initiatives were described where reduced completion rates of paper forms occurred over time, but practice change was sustained.

## 3. Discussion

This study observed that a cellulitis management plan did not improve the appropriateness of antibiotic prescribing for cellulitis at day 1, day 3, or discharge when implemented in three regional health services in Australia. 

The implementation of a cellulitis pathway to improve antibiotic stewardship has been recommended as a practice improvement strategy [8]. There are published examples of the use of a clinical pathway or guideline to positively influence antibiotic prescribing in cellulitis [9,10,11,12]. However, there are notable differences in the approaches described in these earlier studies and the current study that may explain the differences in findings. For example, the current study developed and implemented a cellulitis management plan in collaboration with three independent regional health services, while the other studies were all single-centre studies in large hospitals. When considering antibiotic appropriateness, there were certainly different starting points. For example, the baseline antibiotic appropriateness was higher in the current study (78.7%), with an ambitious goal to increase this to 90% appropriateness. Achieving 90% appropriateness may have, in fact, been a stretch goal, given that few of the common conditions in the NAPS audit reach this. In other studies, the baseline appropriateness or guideline compliance were around 40%, with the intervention increasing this to between 48% and 70% [9,10]. This is well below the achievement of 88.1% in the group commenced on the cellulitis management plan in this study. The cellulitis management plan in the current study was also designed to inform more than just antibiotic prescribing (e.g., treatment of pre-disposing factors), whereas several of the other studies focussed solely on antibiotic choice [9,10].

The real-world uptake of the cellulitis management plan (29.1%) fell below expectations, despite positive feedback on its usefulness. A New Zealand study reported that documented evidence of the use of their cellulitis pathway (part or full) was present in just 13% of patients [10]. This highlights how the uptake of new behaviours in the real world is challenging. The AACTT (Action, Actor, Context, Target, Time) framework was utilised to reflect on potential reasons for sub-optimal uptake for the cellulitis management plan [13]. One identified deficiency was the lack of ownership for completing the cellulitis management plan (Actor). There were differing opinions on who should have the responsibility of documenting each action in the cellulitis management plan. This lack of ownership may have been an unintended consequence of the cellulitis management plan being multidisciplinary. Greater consideration of such behavioural frameworks from the conception of the study may have improved the outcome [14]. 

There are some limitations to the findings of this study. The COVID-19 pandemic decreased hospital activity, and when combined with the low uptake of the cellulitis management plan, the study was underpowered. The small number of cellulitis admissions at the two smaller health services meant that analysis of the data per health service was not feasible. As this was an observational study, the clinical diagnosis and severity of cellulitis could not be verified retrospectively, and clinical cure of cellulitis was not assessed. Confounding factors included different medical staff in the baseline and post-implementation periods. There is no doubt that this study was implemented in extremely difficult circumstances given the COVID-19 pandemic. Local data suggest a reduction in the utilisation of other established clinical resources, such as sepsis pathways, likely due to a preoccupation with COVID-19 requirements. A different outcome may have been achieved in a different set of circumstances. 

Antibiotic prescribing behaviours are known to be complex [15]. Despite implementing a recommended practice-change strategy [8,16], there was no significant change in the appropriateness of antibiotic prescribing for cellulitis. However, this study contributes to the body of knowledge and helps to build a picture of what works in the real world. Further research is required to establish the organisational factors that predict the success of such interventions.

## 4. Materials and Methods

### 4.1. Study Design

This was a hybrid implementation study intended to test the intervention while also gathering information on its feasibility in a real-world setting [17].

### 4.2. Study Setting

Three Victorian health services located in an Australian Statistical Geography Standard Remoteness Area classification [18] of inner or outer regions participated. The health service characteristics are provided in Table S2. 

### 4.3. Intervention

Bundles of care (‘bundles’) are packages of independent evidence-based activities that should be followed for every patient every time [19]. A bundle of care for the management of cellulitis (Table S3) was developed by the research team based on a review of the existing published literature, recommendations from the Australian Therapeutic Guidelines: Antibiotics [7], and cellulitis ‘pathways’ from other organisations. A lower-limb cellulitis management plan (‘cellulitis management plan’) incorporating the bundle components was co-designed with local medical, nursing, and allied health staff to operationalise the bundle elements (Figure S1). Section 3 of the cellulitis management plan provided antibiotic options (including dose and frequency) in the form of a matrix, with consideration of a patient’s allergies, likely pathogen, and location of care. A patient information leaflet was co-designed with consumer input and attached to the cellulitis management plan. 

### 4.4. Implementation

The cellulitis management plan was implemented in the three regional health services in February and March 2020. The launch was preceded and accompanied by face-to-face site-specific education sessions for medical, nursing, and allied health staff that explained the various sections of the cellulitis management plan and other communications (email series, posters, branded notepads) coordinated by the site research officers. 

The primary person responsible for commencing the cellulitis management plan was the admitting medical officer. Completion of the daily review pages was the responsibility of the treating medical team. Completion of the nursing care section (limb elevation, skin care, and patient education) of the daily review pages was the responsibility of the nurse caring for the patient on each shift. Medical, nursing, and allied health staff were requested to provide the patient information leaflet to the patient and discuss the content. 

In March 2020, the first wave of the COVID-19 pandemic impacted Victorian health services, and all non-critical communications to staff were ceased (including those related to this project). In early October 2020, the cellulitis management plan was relaunched with staff across the three health services through a 4-min educative video and associated prize draw. Embedding was again halted by the second wave of COVID-19 in Victoria. 

### 4.5. Evaluation of the Cellulitis Management Plan

The RE-AIM (reach, effectiveness, adoption, implementation, maintenance) framework [20] was adopted to evaluate the cellulitis management plan, utilising both quantitative and qualitative methods. The reach, effectiveness, and implementation components of RE-AIM were most relevant to this study.

### 4.6. Quantitative Component (Reach, Efficacy, Implementation)

#### 4.6.1. Participant Selection

Patients presenting to the three health services between 1 May 2019 and 30 November 2019 (baseline) and between 1 March 2020 to 31 October 2020 (post-implementation) who met the inclusion criterion were identified from the Patient Administration System (PAS). The post-implementation data collection was extended compared to the baseline period to try to achieve statistical power given the reduced hospital activity due to the COVID-19 pandemic and low uptake of the cellulitis management plan. 

The inclusion criterion was adult patients (18 years or over) who were admitted to the hospital (inclusive of parenteral therapies in the home) whose episode of care was assigned the ICD-10-AM coding for cellulitis of the lower limb (L03.13 and L03.14). The medical records of all consecutive patients were screened against the exclusion criteria by site research officers. The exclusion criteria included: patients who were not prescribed an antibiotic, intensive care admission during the episode of care, previous admission for cellulitis in the data collection periods, transfer from another hospital, transfer to another hospital, and where review of medical records by the research officers indicated that the condition was not cellulitis.

#### 4.6.2. Primary and Secondary Outcomes (Efficacy, Reach)

The primary outcome of this study was the percentage of antibiotic prescriptions assessed as appropriate on day 1 of antibiotic therapy before and after the introduction of the cellulitis management plan. This was chosen as the primary outcome because day 1 is reflective of the initial antibiotic choice and a time point that would capture all patients regardless of their length of stay. Secondary outcome measures determined a priori included:

- appropriateness of antibiotic therapy at day 3;
- appropriateness of antibiotic therapy upon discharge;
- time to first antibiotic dose (TFAD);
- duration of antibiotic therapy (total, inpatient, and parenteral therapy);
- length of stay;
- 30-day representation to the same hospital’s Emergency Department with cellulitis;
- 30-day readmission to the same hospital with cellulitis;
- the number of patients who were commenced on a cellulitis management plan;
- the fidelity of the cellulitis management plan completion.

#### 4.6.3. Data Collection

Relevant patient demographics (age, gender, indigenous status) and representation or readmission information were collected from the PAS. A medical record review confirmed the primary reason for admission or representation. Comorbidities known to be associated with cellulitis [21] were manually extracted from the patients’ medical records by research officers (J.B., J.F., M.J., and K.S.). Supplementary codes for chronic conditions (ICD-10-AM codes U78.1 to U88.2) were extracted from coding data to provide a general indication of disease burden in participants. Clinical parameters (temperature, heart rate, respiratory rate, systolic blood pressure, white blood cell count, C-reactive protein, weight, and renal function) were recorded when first available in the patients’ medical records. Allergies to antibiotics were captured from the medical records. 

Antibiotic prescriptions on the National Inpatient Medication Chart were identified and assessed by the research officers. The name, dose, route, frequency, date/time commenced, and date/time ceased of each antibiotic prescribed for cellulitis were recorded, and duration of therapy was calculated. Day 1 and day 3 of antibiotic therapy were defined as the first and third days that the patient received an antibiotic dose as an inpatient. Any route, dose, or frequency changes of the same antibiotic were considered a new order for the analysis. Single (statim) doses did not contribute to the duration of antibiotic therapy calculations. Copies of discharge prescriptions located in the medical records were accessed and the antibiotic name, dose, and quantity prescribed were recorded.

The appropriateness of antibiotic therapy was assessed using the definition from the NAPS [22]. An antibiotic was considered appropriate when it aligned with the recommendations in the Australian Therapeutic Guidelines or local policy, or if it was considered a reasonable alternative for the likely causative pathogen (e.g., directed therapy). For prescriptions deemed inappropriate outside of the agreed parameters, an Infectious Diseases specialist (R.C.) undertook a second (binding) assessment. 

The time to first antibiotic dose (TFAD) was considered the difference between the triage time at the Emergency Department or Urgent Care Centre and the time that the first antibiotic dose was administered. 

Reach (uptake) was determined by the number of eligible patients who had a cellulitis management plan initiated as evidenced by a scanned copy of the cellulitis management plan in the patients’ medical records. Fidelity was assessed by viewing the cellulitis management plan in the patients’ medical records and recording which sections were completed as intended.

#### 4.6.4. Sample Size Estimation for the Primary Outcome and Statistical Analysis

The sample size was calculated based on a dichotomous primary outcome measure with two independent samples (before and after the cellulitis management plan introduction). The following assumptions were applied: α 0.05, β 0.20, baseline rate of inappropriate prescribing of 25% [6], and 15% improvement in the appropriateness of antibiotic prescribing after implementation. The 15% improvement was based on reaching 90% appropriateness, which was the highest appropriateness reached in the NAPS audits for the most common conditions [23]. This resulted in a sample size of 100 patients in each group. 

Data from all three sites were pooled for analysis. The data are presented descriptively as mean and range for normally distributed continuous variables (median, IQR otherwise) and the number and percentage for categorical variables. Student’s *t*-test (two tailed), Mann–Whitney U, and χ^2^ tests were used (where appropriate) to compare baseline and post-implementation data, with a *p*-value less than 0.05 considered statistically significant. For the primary outcome, the full post-implementation sample was compared to the baseline (intention to treat analysis). An analysis of outcomes between those that did and did not commence the cellulitis management plan in the post-implementation period was also undertaken. The Stata statistical package (Version 16.1, StataCorp LLC, College Station, TX, USA) was used for the analysis.

### 4.7. Qualitative Component (Implementation)

The implementation of the cellulitis management plan was further evaluated through key informant interviews. 

A pragmatic sample of five health professionals was selected across medical, nursing, and allied health craft groups at site C using only purposive sampling. A larger sample was intended, but health service workforce constraints due to COVID-19 precluded this. Written consent was obtained from all participants. An interview guide with semi-structured interview questions (see Table S4) was piloted with a health professional (D.B.). Interviews were conducted face to face or via teleconferencing by a study investigator (J.B.) in November 2020. Interviews were audio-recorded and transcribed. Interview transcripts were reviewed and qualitative description was undertaken by a study investigator (J.B.) to describe the respondents’ experiences [24]. 

## Figures and Tables

**Table 1:** Demographics, presenting clinical parameters, and comorbidities of patients included in the baseline and post-implementation samples.

		Baseline (*n* = 165)	Post-Implementation (*n* = 127)	*p*-Value
Demographics				
Gender (%)	Male	102 (61.8%)	82 (64.6%)	0.62
Aboriginal or Torres Strait Islander	Yes	2 (1.2%)	2 (1.6%)	0.77
Age at admission [mean years] (range)		64 (18–97)	60 (20–97)	0.08
Length of stay [median] (IQR)		3.00 (1–6)	3.76 (1–5)	0.72
ICD-10-AM code	L03.13 P L03.13 C L03.14 P L03.14 C	127 (77.0%) 13 (7.9%) 24 (14.5%) 1 (0.6%)	102 (80.3%) 10 (7.9%) 14 (11.0%) 1 (0.8%)	0.50 1.00 0.39 0.84
Diagnosis-Related Group	J64A J64B Other	43 (26.1%) 82 (49.7%) 40 (24.2%)	29 (22.8%) 65 (51.2%) 33 (26.0%)	0.51 0.80 0.73
Presenting clinical parameters from initial presentation or when first available in the medical record				
Temperature [mean °C] (range)		36.6 (34.1–39.5)	36.9 (35.2–39.9)	0.02
Heart rate [mean beats per min] (range)		88 (55–142)	94 (50–180)	<0.01
Respiratory rate [mean breaths per min] (range)		18 (12–40)	19 (12–48)	0.03
Systolic blood pressure [mean mmHg] (range)		132 (65–194)	135 (100–198)	0.20
WBC [mean cells/L] (range)		10.5 (2.5–38.9) *n* = 157	11.1 (1.2–29.1) *n* = 122	0.29
CRP [mean mg/L] (range)		67 (1–410) *n* = 145	74 (0.7–458) *n* = 118	0.49
Weight [mean kg] (range)		103 (42–190) *n* = 62	99.6 (47–220) *n* = 73	0.54
eGFR greater than 45 mL/min		132 (84.6%) *n* = 156	102 (82.3%) *n* = 124	0.50
Number of patients with predisposing factors known to be associated with cellulitis				
None		64 (38.8%)	44 (34.6%)	0.48
Obesity		44 (26.7%)	36 (28.3%)	0.85
Diabetes mellitus		43 (26.1%)	38 (29.9%)	0.45
Renal disease (eGFR below 45 mL/min)		24 (14.5%)	22 (17.3%)	0.64
Congestive heart failure		22 (13.3%)	16 (12.6%)	1.00
Chronic obstructive pulmonary disease		19 (11.5%)	12 (9.4%)	0.41
Lymphoedema		17 (10.3%)	12 (9.4%)	0.77
Liver disease		10 (6.1%)	10 (7.9%)	0.50
Coronary artery bypass grafting		10 (6.1%)	2 (1.6%)	0.09
Tinea pedis		6 (3.6%)	3 (2.4%)	0.33
Varicose veins		3 (1.8%)	2 (1.6%)	1.00
Hemiplegia/paraplegia		1 (0.6%)	3 (2.4%)	0.48
Number of patients with common chronic conditions ^#^				
Hypertension		65 (39.4%)	44 (38.3%)	0.85
Depression		33 (20.0%)	13 (11.3%)	0.04
Arthritis and osteoarthritis		28 (17.0%)	23 (20.0%)	0.53
Obesity		24 (14.5%)	19 (16.5%)	0.65
Ischaemic heart disease		23 (13.9%)	10 (8.7%)	0.17
Chronic obstructive pulmonary disease		15 (9.1%)	9 (7.8%)	0.70

L03.13 = lower-limb cellulitis, L03.14 = cellulitis of foot, P = primary condition, C = complication. J64A: cellulitis with catastrophic or severe complications and comorbidities, J64B: cellulitis without catastrophic or severe complications and comorbidities. ^#^ Data not available for 12 patients at site B due to coding differences (*n* = 115).

**Table 2:** Prescriptions assessed as appropriate using the NAPS definitions at day 1, day 3, and discharge at baseline and post-implementation of the cellulitis management plan.

	Prescriptions Assessed as Appropriate	95% CI	*p*-Value
Day 1			
Baseline	78.7% (144/183)	72.1–84.1%	0.47
Post-implementation	81.8% (126/154)	74.8–87.2%	
Commenced plan	88.1% (37/42)	73.9–95.1%	0.22
Without plan	79.5% (89/112)	70.9–86.0%	
Day 3			
Baseline	87.8% (108/123)	80.7–92.6%	0.50
Post-implementation	90.9% (90/99)	83.3–92.6%	
Commenced plan	96.0% (24/25)	74.5–99.5%	0.31
Without plan	89.2% (66/74)	79.6–94.6%	
Discharge			
Baseline	85.6% (101/118)	77.9–90.9%	0.09
Post-implementation	92.6% (100/108)	85.8–96.3%	
Commenced plan	97.2% (35/36)	81.7–99.6%	0.19
Without plan	90.3% (65/72)	80.8–95.3%	

Commenced plan: those who had a cellulitis management plan in their medical record at the time of review. Without plan: those who did not have a cellulitis management plan in their medical record at the time of review.

**Table 3:** Most common antibiotics prescribed on day 1 of inpatient antibiotic therapy.

Baseline (*n* = 183)		Post-Implementation (*n* = 154)	
Flucloxacillin ^#^	83 (45.4%)	Flucloxacillin ^#^	70 (45.5%)
Cefazolin	66 (36.1%)	Cefazolin	49 (31.8%)
Piperacillin/tazobactam	9 (4.9%)	Benzylpenicillin	8 (5.2%)
Clindamycin ^#^	8 (4.4%)	Clindamycin ^#^	8 (5.2%)
Cefalexin	7 (3.8%)	Piperacillin/tazobactam	5 (3.2%)

^#^ IV and oral orders combined.

**Table 4:** Secondary outcome measures.

Outcome	Group	Result	95% CI	*p*-Value
Presented to Emergency Department or Urgent Care Centre with cellulitis within 30 days of discharge from hospital for a cellulitis-related episode of care	Baseline	11/165 (6.7%)	3.7–11.7%	0.90
	Post-implementation	8/127 (6.3%)	3.2–12.2%	
	Commenced plan	2/37 (5.4%)	1.3–20.0%	0.79
	Without plan	6/90 (6.7%)	3.0–14.2%	
Readmitted with cellulitis within 30 days of discharge	Baseline	8/165 (4.8%)	2.4–9.4%	0.42
	Post-implementation	9/127 (7.1%)	3.7–13.1%	
	Commenced plan	2/37 (5.4%)	1.3–20.0%	0.80
	Without plan	7/90 (7.8%)	3.7–15.6%	
Median acute length of stay [days] (IQR)	Baseline	3.00 (1–6)	2.0–4.0	0.72
	Post-implementation	3.00 (1–5)	2.0–4.0	
	Commenced plan	3.00 (1–4)	1.1–3.9	0.28
	Without plan	3.00 (1–5)	2.0–4.0	
Median time to first antibiotic dose [min] (IQR)	Baseline (*n* = 158)	192 (121–329)	177.0–221.8	<0.01
	Post-implementation (*n* = 124)	140 (85–249)	116.1–176.4	
	Commenced plan (*n* = 36)	138 (96–247)	106.2–202.4	0.64
	Without plan (*n* = 88)	140 (85–249)	108.6–180.9	
Median total duration of antibiotic therapy (inpatient and discharge) [hours] IQR)	Baseline	168 (132–212)	162.0–176.6	0.95
	Post-implementation	168 (135–207)	157.0–174.0	
	Commenced plan	168 (152–180)	155.1–174.0	0.93
	Without plan	168 (133–213)	154.0–179.4	
Median inpatient antibiotic therapy duration (hours) (IQR)	Baseline	54 (23–109)	42.7–63.2	0.46
	Post-implementation	51 (24–88)	39.0–60.0	
	Commenced plan	38 (18–60)	26.2–55.9	0.13
	Without plan	55 (24–97)	42.2–72.0	
Median IV antibiotic therapy duration (hours) (IQR)	Baseline	42 (14–73)	30.4–48.0	0.93
	Post-implementation	42 (12–72)	31.0–49.1	
	Commenced plan	35.5 (16–60)	23.4–50.2	0.55
	Without plan	43 (12–81)	31.3–56.4	
Median duration of antibiotics prescribed at discharge (hours) (IQR)	Baseline (*n* = 115)	144 (120–144)	120.0–144.0	0.08
	Post-implementation (*n* = 107)	120 (120–144)	120.0–128.0	
	Commenced plan (*n* = 35)	120 (120–144)	120.0–139.5	0.73
	Without plan (*n* = 72)	120 (120–144)	120.0–143.0	

Baseline (*n* = 165), post-implementation (*n* = 127), commenced plan (*n* = 37), without plan (*n* = 90), except where specified. Commenced plan: those who had a cellulitis management plan in their medical record at the time of review. Without plan: those who did not have a cellulitis management plan in their medical record at the time of review. Time to first antibiotic dose: difference between the triage time at the Emergency Department or Urgent Care Centre and the time that the first antibiotic dose was administered.

**Table 5:** Quotes from the key informant interviews grouped into themes.

Usefulness of the cellulitis management plan
*So I think it certainlyhas helped in terms of providing, particularly for our juniors and particularly some of our overseas train docs, a working tool that they can actually use at the time to make sure that they are prescribing those antibiotics and stuff correctly. [P2, ED Consultant, interview]* *Really clear pathways. Fantastic for junior and senior staff alike. [M1, Medical Officer, survey]* *Good overall cellulitis plan for team and probably good to ensure that what is communicated on the round from the consultant is backed up and followed. Good guide for ensuring all risks are managed appropriately. [M3, Medical Officer, survey]* *I think where it sort of stands on its own with a lot of the other pathways and things in the hospital, is that it does actually include a very structured and well thought out patient education component as well, which is often lacking. [P3, Pharmacist, interview]* *Have been nursing for over 20 years, aware how to look after lower limb cellulitis without a chart to tell me how—have been doing these interventions long before a chart was created. [N7, Nurse, survey]*
Impact of the cellulitis management plan on practice
*I’ve learned stuff myself through havingused the form and particularly discussing the issue about those particular sort of specific patient cohorts or sets that you choose to use the benzylpenicillin for. [P2, ED Consultant, interview]* *I think prior to, or at least in medical school, cellulitis is treated with fluclox or Keflex. But I think the situation here, at least for mild cellulitis, is benzylpenicillin. Which was sort of eye opening for me, I suppose. But it is also reflected in the other therapeutic guidelines. So I did change prescribing practice. [P1, ED Consultant, interview]* *The cellulitis plan hasn’t greatly changed my practice but I would think for more junior doctors it would be very helpful. [M2, Medical Officer, survey]* *A pathway policy exists for a reason. I just tried to adhere to it when I do my rounds. [P1, ED Consultant, interview]* *Although I don’t think I recall seeing any cellulitis pathways in patients’ histories, what I do notice that a lot of the secondary sort of aspects of care. So, tinea being treated and things like that. [P3, Pharmacist, interview]*
Challenges
*It’s actually those first two pages of that bundle, I think, are actually more clinically relevant/clinically useful to our medical staff then perhaps some of the tick boxes and stuff, towards the end… I think that having that first page or two as a working tool is probably where the money is in terms of the clinical utility. [P2, ED Consultant, interview]* *So the first couple of pages of the form from at least from a medical perspective are fairly self-explanatory. It tells you, it’s very easy, it tells you what to prescribe. And that’s quite straightforward. And so, I thought that the first couple of pages fairly easy to use. So just the front of the form was quite easy and towards the end of the form perhaps clunky. [P1, ED Consultant, interview]* *Bulky format. [N1, Nurse, survey]* *From experience treating lower leg cellulitis patients I’ve found the pathway too cumbersome; most ED staff don’t fill it out correctly. If it could be condensed with basic info it would probably be used more. The progress review section on each day doesn’t really get used and could be condensed. [N8, Nurse, survey]* *Usage has dropped off. Good uptake by [ward name] originally if requested completion, now nil completion. [N1, Nurse, survey]*
Responsibility for completing the cellulitis management plan
*I suppose, with the sepsis pathway, it was designed for the nursing staff to be able to command it and put it on to the doctor’s nose. I think we had more success working that way around and then we would have if the doctors were to be the ones required initiate. With [this] I suppose it, given that there is much more emphasis on actually making that clinical diagnosis excluding other sort of differentials sort of early on in the piece, it does sort of fit more so with it being in front of the doctor when they’re doing their admission to do with the patient. [P3, Pharmacist, interview]* *I think, personally, I think the doctor should definitely be the one instigating the form. A lot of these things fall onto the nurses, because without the nurses, they don’t happen. [P4, Wound Care Consultant, interview]* *Other places where I work, the responsibility for filling out the [similar form], if it’s using the antibiotics, actually falls to the ED pharmacist*… *So there is, I suppose because have more resources, higher adherence to the pathway. [P1, ED Consultant, interview]* *Well within the scope of a pharmacist to start this but they’re not a 24-h presence in the hospital. [P3, Pharmacist, interview]*
Familiarity with the content of the resource over time
*I don’t know whether you’ve seen this or noticed this with the sepsis pathway for example as well. It’s once everyone’s familiarized themselves with it and they know exactly what’s on there, know the different antibiotic choices in different situations. Since people become so familiar with the contents of it that then they can fatigue as well, particularly when you’ve got sick patient or you’ve got multiple different patient priorities and stuff at the same time. [P2, ED Consultant, interview]* *Although we know that people have gotten out of the habit of using [the sepsis pathway], there hasn’t been like a significant drop off in a lot of those sort of management aspect that were initially not done*… *Yeah, it’s one of those things that even though that the use of the pathway has trailed off, you are still seeing aspects of that pathway being brought through in people’s practice. [P3, Pharmacist, interview]*

## Data Availability

The data that support the findings of this study are available from the corresponding author upon reasonable request.

## Supplementary Materials

The following are available online at https://www.mdpi.com/article/10.3390/antibiotics10111288/s1: Table S1: Reasons why antibiotic prescriptions for cellulitis were assessed as inappropriate, Table S2: Characteristics of the three participating health services who implemented the cellulitis management plan, Table S3: The components of the cellulitis bundle of care that formed the cellulitis management plan, Table S4: Semi-structured interview questions, Figure S1: Adult lower-limb cellulitis management plan.
