# Supplementary material for: Implementation of a Cellulitis Management Plan in Three Australian Regional Health Services to Address an Evidence–Practice Gap in Antibiotic Prescribing
Source: Antibiotics (Basel). 2021 Oct 22;10(11):1288. doi: 10.3390/antibiotics10111288 (PMC8615114; doi:10.3390/antibiotics10111288)
Supplement: Supplementary file 1 [file antibiotics-10-01288-s001.zip › Figure_S1.pdf]

COPY ONLY - NOT FOR CLINICAL USE

[redacted]

Adult Lower Limb Cellulitis

Confirm diagnosis (considering differential diagnoses) - pg 1
Evaluate severity (assess for danger signs) - pg 1
Location of care and antibiotic choice - pg 2
Limb elevation plan - pg 3
Look for predisposing factors - pg 3
Intravenous to oral switch - pg 4 onwards
Treatment duration review - pg 4 onwards

U.R. Number ______________________
Surname ______________________
Given Names ______________________
D.O.B. / / Sex ______________________

Attach patient ID Labels to all pages of this form before commencing any documentation.

1. Confirm diagnosis considering differential diagnoses

Suspect cellulitis when there is acute onset of 2 or more of:

- Heat
- Pain
- Redness
- Swelling

Cellulitis is usually unilateral in the lower leg. Reconsider diagnosis if bilateral (see differential diagnosis). Fever, chills, sweats or rigors may be present in some patients. Non-specific symptoms (such as lethargy, anorexia) may also be present.

Differential diagnosis

Deep vein thrombosis: important to consider– an ultrasound may be required to exclude.

Venous stasis/ lipodermatosclerosis: common and should be considered especially if bilateral.

Dermatitis or vasculitis: rarer, but skin changes can mimic cellulitis.

Septic joint or gout: consider if skin changes are overlying joints.

Contact dermatitis: consider if exposed to irritants.

Charcot neuroarthropathy (foot): consider if diabetic / elevated HbA1c and neuropathy present

IMPORTANT

Erythema can spread for up to 72 hours after effective treatment for cellulitis has commenced.

If the person feels well and is otherwise improving, there is often no need to change antibiotics. Reassurance, continuation of antibiotic therapy and close clinical monitoring may be appropriate.

If there are features of clinical deterioration (increased pain, ongoing rigors): seek ID advice.

2. Evaluate severity and assess for danger signs

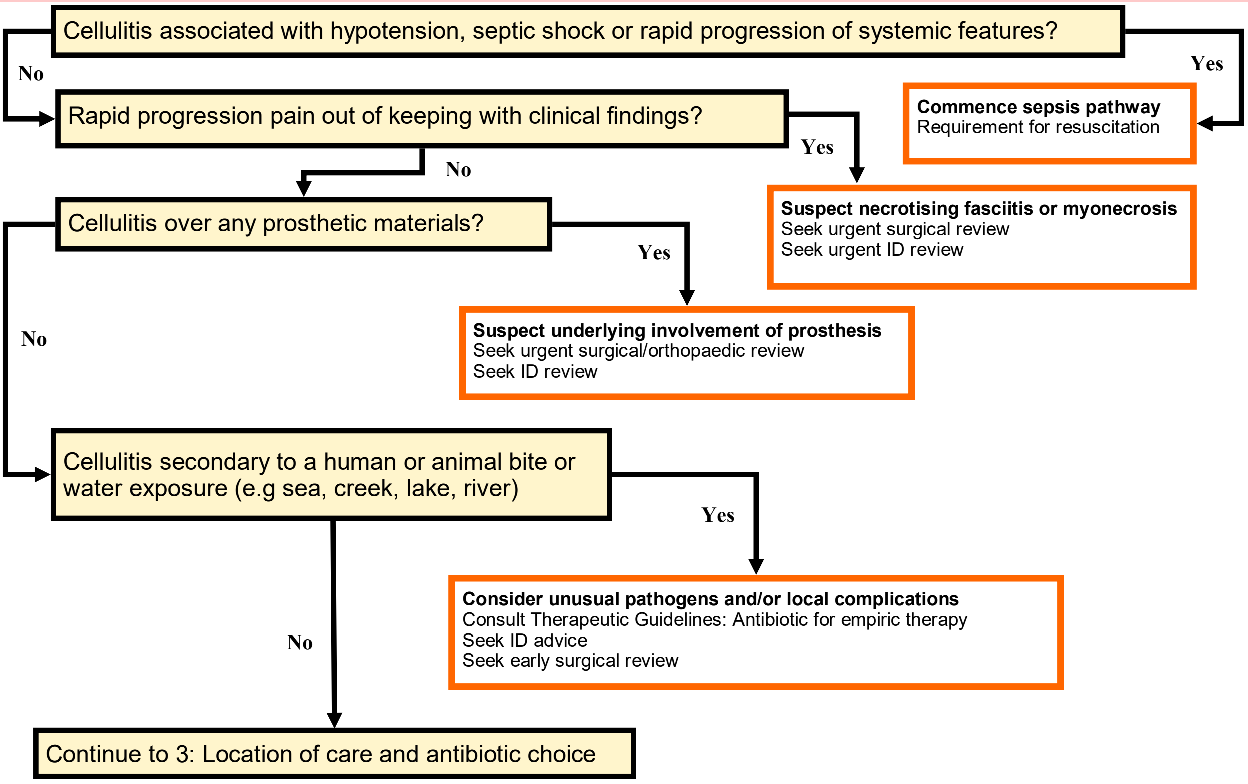

Tool developed by the CELLLIT Project, [redacted]

[redacted]

Adult Lower Limb Cellulitis

Confirm diagnosis (considering differential diagnoses) - pg 1
Evaluate severity (assess for danger signs) - pg 1
Location of care and antibiotic choice - pg 2
Limb elevation plan - pg 3
Look for predisposing factors - pg 3
Intravenous to oral switch - pg 4 onwards
Treatment duration review - pg 4 onwards

U.R. Number ______________________
Surname ______________________
Given Names ______________________
D.O.B. / / Sex ____________

AFFIX PATIENT LABEL HERE

3 . Location of care and antibiotic choice

a) Location of care (circle)

HOME	SSU	HITH	Ward
Systemically well<br>**AND**<br>No other comorbidities requiring inpatient care<br>**AND**<br>Able to tolerate oral therapy<br>**AND**<br>Able to self manage antibiotic therapy and self-care	See MR/266.07 Pathway (SSU) Cellulitis	See MR/360.5 HITH Consent and Assessment Form	Not suitable for care at home, in SSU or HITH

Complete **baseline investigations:** FBE, U&E, BSL and CRP

Consider
Blood culture: if systemically unwell (tachycardia, tachypnoea, febrile) or clinically concerned
Swab or tissue biopsy: only if open wound or infected ulcer **cleanse area before swab/biopsy*

b) Antibiotic choice (circle & prescribe on the medication chart)

Assess for penicillin allergy: clarify the nature of the allergy and de-label allergy if appropriate.
Assess whether risk factors for *S. aureus* are present e.g. focal abscess or boil, traumatic wound, ulcer or exudative wound.
Prescribe chosen antibiotic on medication chart and ensure timely administration of first dose.

	No penicillin allergy	No penicillin allergy	Mild and non-immediate penicillin allergy	Severe or immediate penicillin allergy
	No risk factors for Staph aureus	Risk factors for *Staph aureus*		
**Outpatient**	Phenoxymethylpenicillin 500mg-1g orally 6 hourly	Flucloxacillin 500mg-1g orally 6 hourly	Cefalexin 500mg-1g orally 6 hourly	Clindamycin 450mg orally 8 hourly
**Short stay unit**	IV: Benzylpenicillin 1.2 g IV 6 hourly and assess daily for oral switch<br><br>Oral: Phenoxymethylpenicillin 500mg-1g orally 6 hourly	IV: Flucloxacillin 2g IV 6 hourly and assess daily for oral switch<br><br>Oral: Flucloxacillin 500mg-1g orally 6 hourly	IV: Cefazolin 2g IV 8 hourly and assess daily for oral switch<br><br>Oral: Cefalexin 1g orally 6 hourly	IV: Clindamycin 600mg IV 8 hourly and assess daily for oral switch<br><br>Oral: Clindamycin 450mg orally 8 hourly
**HITH**	Cefazolin 2g IV 12 hourly<br>OR<br>Cefazolin 2g IV daily with 1g probenecid daily<br>OR<br>If PICC line: flucloxacillin 8 to 12 g IV by 24-hour continuous infusion		Cefazolin 2g IV 12 hourly<br>OR<br>Cefazolin 2g IV daily with 1g probenecid daily	Vancomycin infusion may be an option – consult with pharmacy
**Inpatient**	IV: Benzylpenicillin 1.2 g IV 6 hourly and assess daily for oral switch<br><br>Oral: Phenoxymethylpenicillin 500mg-1g orally 6 hourly	IV: Flucloxacillin 2g IV 6 hourly and assess daily for oral switch<br><br>Oral: Flucloxacillin 500mg-1g orally 6 hourly	IV: Cefazolin 2g IV 8 hourly and assess daily for oral switch<br><br>Oral: Cefalexin 1g orally 6 hourly	IV: Clindamycin 600mg IV 8 hourly and assess daily for oral switch<br><br>Oral: Clindamycin 450mg orally 8 hourly

Renal impairment

Flucloxacillin:	CrCl less than 10 mL/min: 1g IV 6 hourly / 500mg orally 8 hourly
Cefazolin:	CrCl 20–40 mL/min: 1g IV 8 hourly or 2g IV 12 hourly<br>CrCl less than 20 mL/min: 0.5g IV 12 hourly or 1g IV daily
Cefalexin:	CrCl less than 10mL/min: 500mg orally 8 hourly
Benzylpenicillin	CrCl 10-50mL/min: 900mg IV 6 hourly<br>CrCl less than 10mL/min: 600mg IV 6 hourly

No adjustment required: phenoxymethylpenicillin & clindamycin

Dosage range for oral antibiotics
Consider using the higher dose for otherwise well patients who are under 75 years AND over 50kg

Weight 120kg or more:
3g of cefazolin per dose
If renally impaired, reduce dose by 50%

COPY ONLY - NOT FOR CLINICAL USE

[redacted]

COPY ONLY - NOT FOR CLINICAL USE

[redacted]

**Adult Lower Limb Cellulitis**	U.R. Number ____________
Confirm diagnosis (considering differential diagnoses) - pg 1	Surname ____________
Evaluate severity (assess for danger signs) - pg 1	Given Names ____________
Location of care and antibiotic choice - pg 2	D.O.B. / / Sex ____________
Limb elevation plan - pg 3	
Look for predisposing factors - pg 3	
Intravenous to oral switch - pg 4 onwards	
Treatment duration review - pg 4 onwards	

Attach patient ID Labels to all pages of this form before commencing any documentation.

4. Limb elevation: management plan

MEDICAL OFFICER TO TICK INSTRUCTIONS

☐ Full elevation with bed rest **[prescribe DVT prophylaxis unless contraindicated]**

☐ Elevation at rest **[consider DVT prophylaxis]**

5. Look for predisposing factors

MEDICAL OFFICER TO TICK THOSE IDENTIFIED & ACTION

Cellulitis due to	SUGGESTED PLAN
☐ Tinea pedis	Terbinafine 1% topically 12 hourly for 14 days<br>Consider outpatient referral to podiatry if deep fissures#
☐ Wound	Cleanse area then take wound swab or biopsy<br>Check wound care referral criteria<br>If wound is on foot, consider outpatient referral to High Risk Foot Clinic#
☐ Lymphoedema or venous insufficiency with oedema (no wound)	Refer to the lymphoedema clinic#
☐ Obesity	Consider referral to the BHS Healthy Weight Management Program#
☐ Poorly controlled diabetes	Refer to *Inpatient Blood Glucose Level Management In Adult Diabetes Patients - CPP0594*<br>Consider referral to endocrinologist (medical management), diabetes educator or dietitian (education) or podiatry (foot care).
☐ Immunosupression	Review frequently for deterioration, ensure blood cultures, look for underlying cause and seek ID advice
☐ Other	

#Refer via Central Intake: referrals will be accepted via fax (5320 3893) or e-referral on the Service Coordination Tool Template (SCTT) and/or the Victorian Statewide Referral Form (VSRF).

☐ Patient leaflet provided

Notes

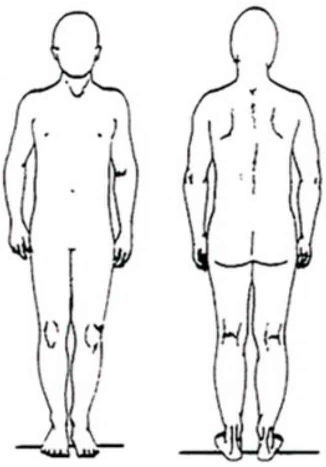

Signature, print name, designation and date

UR No:__________ Surname:__________________ Given Names:__________________ DOB:__________ Sex:____

Date: —/—/—— **Review page number** 1

Record the patient's review each day by ticking box. If not applicable, write N/A in the box.

PROGRESS REVIEW	Description	Reviewed (Tick box)
**Pending investigations checked**	Wound swabs/biopsy<br>Blood cultures<br>Other	
**Management plan review**	**Limb elevation (TICK PLAN)**<br>☐ Full elevation **[prescribe DVT prophylaxis unless contraindicated]**<br>☐ Elevation at rest **[consider DVT prophylaxis]**	
	**Predisposing factors actions**<br>1.<br>2.<br>3.	
**Intravenous to oral antibiotic switch**	**Local symptoms can worsen for up to 72 hours after effective therapy starts. Switching to oral antibiotics is still appropriate if:**<br>☐ Clinical improvement evident<br>☐ Fever resolved or improving<br>☐ No unexplained haemodynamic instability<br>☐ Tolerating oral intake with no concerns about malabsorption<br><br>**Oral therapy appropriate (circle)** Y N<br><br>**Comment**	
**Treatment duration review**	Total duration of therapy (intravenous plus oral) **is five to ten days**<br><br>CURRENT DURATION OF THERAPY (COMPLETE DAYS RECEIVED)<br>IV ☐ + ORAL ☐ = TOTAL ☐	
**Patient education**		

Signature, print name, designation and date

NURSING CARE INITIAL WHEN CHECKED/ COMPLETED	Morning	Afternoon	Evening
**Limb elevation** as per management plan			
**Skin care attended:** wash, moisturise & apply barrier (or follow wound care instructions)			
**Patient education**			

NOTES/COMMENTS/VARIANCES

COPY ONLY - NOT FOR CLINICAL USE

UR No:__________ Surname:_________________ Given Names:________________ DOB:___________ Sex:____

Date: —/—/—— **Review page number:** 2

Record the patient's review each day by initialing each box.
If not applicable, write N/A in the box.

PROGRESS REVIEW	Description	Reviewed (Tick box)
**Pending investigations checked**	Wound swabs/biopsy<br>Blood cultures<br>Other	
**Management plan review**	**Limb elevation (TICK PLAN)**<br>☐ Full elevation **[prescribe DVT prophylaxis unless contraindicated]**<br>☐ Elevation at rest **[consider DVT prophylaxis]**	
	**Predisposing factors actions**<br>1.<br>2.<br>3.	
**Intravenous to oral antibiotic switch**	**Local symptoms can worsen for up to 72 hours after effective therapy starts. Switching to oral antibiotics is still appropriate if:**<br>☐ Clinical improvement evident<br>☐ Fever resolved or improving<br>☐ No unexplained haemodynamic instability<br>☐ Tolerating oral intake with no concerns about malabsorption<br><br>**Oral therapy appropriate (circle)** **Y** **N**<br><br>**Comment**	
**Treatment duration review**	Total duration of therapy (intravenous plus oral) **is five to ten days**<br><br>**CURRENT DURATION OF THERAPY (COMPLETE DAYS RECEIVED)**<br>**IV** ☐ + **ORAL** ☐ = **TOTAL** ☐	
**Patient education**		

Signature, print name, designation and date

**NURSING CARE INITIAL WHEN CHECKED/ COMPLETED**	**Morning**	**Afternoon**	**Evening**
**Limb elevation** as per management plan			
**Skin care attended:** wash, moisturise & apply barrier (or follow wound care instructions)			
**Patient education**			

NOTES/COMMENTS/VARIANCES

COPY ONLY - NOT FOR CLINICAL USE

Date: —/—/—— **Review page number** 3 Record the patient's review each day by ticking box. If not applicable, write N/A in the box.

PROGRESS REVIEW	Description	Reviewed (Tick box)
**Pending investigations checked**	Wound swabs/biopsy<br>Blood cultures<br>Other	
**Management plan review**	**Limb elevation (TICK PLAN)**<br>☐ Full elevation **[prescribe DVT prophylaxis unless contraindicated]**<br>☐ Elevation at rest **[consider DVT prophylaxis]**	
	**Predisposing factors actions**<br>1.<br>2.<br>3.	
**Intravenous to oral antibiotic switch**	**Local symptoms can worsen for up to 72 hours after effective therapy starts. Switching to oral antibiotics is still appropriate if:**<br>☐ Clinical improvement evident<br>☐ Fever resolved or improving<br>☐ No unexplained haemodynamic instability<br>☐ Tolerating oral intake with no concerns about malabsorption<br><br>**Oral therapy appropriate (circle)** **Y** **N**<br><br>**Comment**	
**Treatment duration review**	Total duration of therapy (intravenous plus oral) **is five to ten days**<br><br>CURRENT DURATION OF THERAPY (COMPLETE DAYS RECEIVED)<br>**IV** ☐ + **ORAL** ☐ = **TOTAL** ☐	
**Patient education**		

Signature, print name, designation and date

NURSING CARE INITIAL WHEN CHECKED/ COMPLETED	Morning	Afternoon	Evening
**Limb elevation** as per management plan			
**Skin care attended:** wash, moisturise & apply barrier (or follow wound care instructions)			
**Patient education**			

NOTES/COMMENTS/VARIANCES

COPY ONLY - NOT FOR CLINICAL USE

[redacted]

Cellulitis

(sell-u-ly-tiss)

1. What is cellulitis?

Cellulitis is a skin infection that often occurs in the legs. The area is usually:

- **Warm**
- **Red**
- **Sore**
- **Tight**

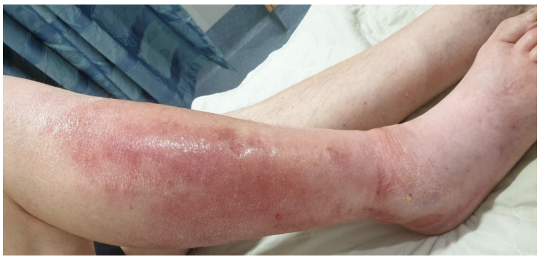

You may also have other signs of infection such as fever, sweats, shakes or loss of appetite.

Cellulitis happens when bacteria enters the inner layers of your skin, often when it is damaged or broken (e.g. from an insect bite, tinea or when the skin is cracked and dry).

2. What should I expect when I am in hospital?

Antibiotics to treat the infection
via a drip or as a tablet/capsule

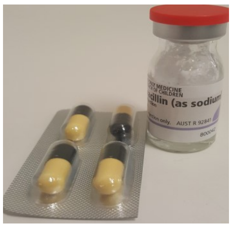

Washing of the limb

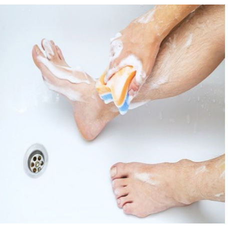

Elevation of the limb
to reduce swelling

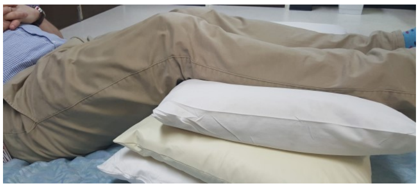

Treatment for other conditions which can help the symptoms of cellulitis improve:

e.g. tinea

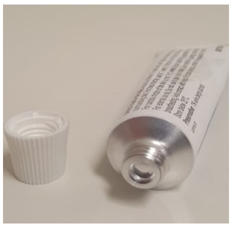

dry skin

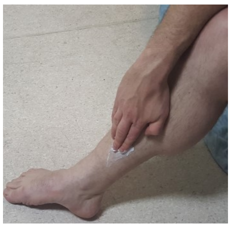

compression to reduce swelling

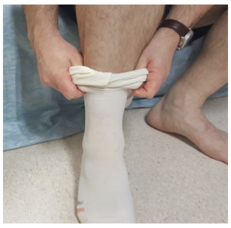

REMEMBER: the area of redness may spread, even once you start antibiotics.
It can take weeks or months for the skin changes caused by cellulitis to disappear.
In some people, the skin changes are permanent.

My questions:

[redacted]

Cellulitis

(sell-u-ly-tiss)

3. What care is required for my cellulitis at home?

If you’ve had cellulitis before, you are more likely to have it again.
These tips may help prevent it returning.

Elevate the limb as much as possible
1. while the area is still red, hot and swollen
2. if the limb becomes swollen again

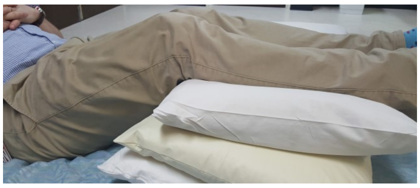

Continue to take the antibiotics as prescribed for you

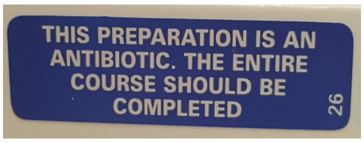

Wash or bathe daily
Use a soap free, pH adjusted cleanser
(ask your pharmacist for suggestions)

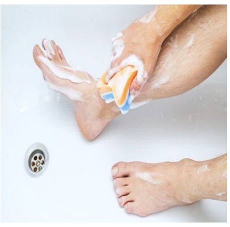

Moisturise your skin at least daily
to prevent it becoming dry and cracked.
Use a perfume free moisturiser.

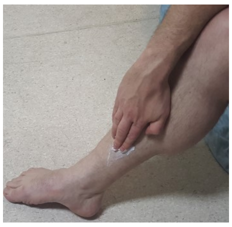

Check feet and toes often for fungal infections
(e.g. tinea, Athlete’s foot) and treat if found

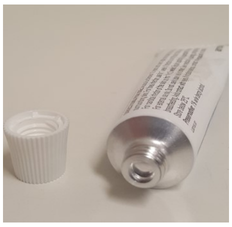

Maintain a healthy weight

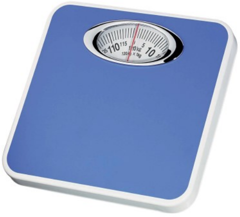

4. When should I seek medical advice?

Seek medical advice if:

- the redness continues to spread very quickly
- you develop a high fever or sweats
- the area becomes very painful
- the area develops pus

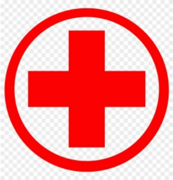

CELLLIT Project
Version 2 January 2020
[redacted]

5. More information?

These websites may be of interest
DermNet NZ: www.dermnetnz.org/topics/cellulitis/
Better Health Channel: www.betterhealth.vic.gov.au (search for cellulitis)

Disclaimer: While we make every effort to make sure the information in this leaflet is accurate and informative, the information does not take the place of professional or medical advice. We do not accept any liability for any injury, loss or damage caused by use of the information.
