# Supplementary material for: Implementation of a Cellulitis Management Plan in Three Australian Regional Health Services to Address an Evidence–Practice Gap in Antibiotic Prescribing
Source: Antibiotics (Basel). 2021 Oct 22;10(11):1288. doi: 10.3390/antibiotics10111288 (PMC8615114; doi:10.3390/antibiotics10111288)
Supplement: Supplementary file 1 [file antibiotics-10-01288-s001.zip › Table_S1.pdf]

**DAY 1**			**Post implementation**		
		**Baseline**	**All**	**With plan**	**Without plan**
**Reason for the prescription being assessed as inappropriate**#	Allergy mismatch	3	0	0	0
	Incorrect antibiotic	4	5	1	4
	Incorrect dose	27	15	3	12
	Incorrect frequency	5	5	1	4
	Non guideline compliant	1	4	1	3
	Incorrect route	0	1	1	0
	Serious drug interaction	0	0	0	0
**Day 3**			**Post implementation**		
		**Baseline**	**All**	**With plan**	**Without plan**
**Reason for the prescription being assessed as inappropriate**#	Allergy mismatch	1	0	0	0
	Incorrect antibiotic	4	2	0	2
	Incorrect dose	9	3	0	3
	Incorrect frequency	4	3	1	2
	Non guideline compliant	0	1	0	1
	Incorrect route	0	0	0	0
	Serious drug interaction	0	0	0	0
**Discharge**			**Post implementation**		
		**Baseline**	**All**	**With plan**	**Without plan**
**Reason for the prescription being assessed as inappropriate**#	Allergy mismatch	0	0	0	0
	Incorrect antibiotic	4	3	0	3
	Incorrect dose	7	3	0	3
	Incorrect frequency	5	2	1	1
	Non guideline compliant	2	0	0	0
	Incorrect route	0	0	0	0

Note that each patient may have been prescribed multiple antibiotics on Day 1 or Day 3 and have multiple inappropriate reasons.

S1 Reasons why antibiotic prescriptions for cellulitis were assessed as inappropriate
