# Supplementary material for: Implementation of a Cellulitis Management Plan in Three Australian Regional Health Services to Address an Evidence–Practice Gap in Antibiotic Prescribing
Source: Antibiotics (Basel). 2021 Oct 22;10(11):1288. doi: 10.3390/antibiotics10111288 (PMC8615114; doi:10.3390/antibiotics10111288)
Supplement: Supplementary file 1 [file antibiotics-10-01288-s001.zip › Table_S2.pdf]

Characteristic	Site A	Site B	Site C
Hospital type	Local	Sub-regional	Regional
Remoteness classification	Inner regional	Outer regional	Inner regional
Bed numbers (acute & sub-acute)	37	89	355
Medical staff structure	GP led	Salaried staff	Salaried staff
AMS pharmacist	Yes	Yes	Yes
On-site ID specialist	No	No	Yes

Table S2 Characteristics of the three participating health services who implemented the cellulitis management plan
