# Supplementary material for: Implementation of a Cellulitis Management Plan in Three Australian Regional Health Services to Address an Evidence–Practice Gap in Antibiotic Prescribing
Source: Antibiotics (Basel). 2021 Oct 22;10(11):1288. doi: 10.3390/antibiotics10111288 (PMC8615114; doi:10.3390/antibiotics10111288)
Supplement: Supplementary file 1 [file antibiotics-10-01288-s001.zip › Table_S4.pdf]

1. Please outline your understanding of the aims of the ‘cellulitis project’ (known as CELLLIT) that introduced the cellulitis management plan and created the consumer leaflet.
2. How do the tools developed in this project compare to other tools available in your setting?
   If required, ask directly:
   a. What advantages did the cellulitis management plan offer compared to other tools?
   b. What disadvantages did the cellulitis management plan offer compared to other tools?
3. Thinking back to when the cellulitis management plan was introduced, how prepared did you feel to use the cellulitis management plan as intended? Why?
4. How has the cellulitis management plan influenced cellulitis management in your organisation?
5. Describe whether you think the cellulitis management plan should be made available to other organisations to use. Why do you say that?

5b If you think the cellulitis management plan should be made available to other organisations to use, outline:
   a. Whether further pilot testing would be required?
   b. What components should be kept the same?
   c. What components should be changed?

6. How confident are you that the cellulitis management plan will still be in use in 12 months’ time? Why do you say this?
7. Would you like to add anything else to our discussion about the cellulitis management plan introduced by the CELLLIT project?

Table S4. Semi-structured interview questions
